# Ethics, regulation, and beyond: the landscape of research with pregnant women

**DOI:** 10.1186/s12978-017-0421-3

**Published:** 2017-12-14

**Authors:** Carla Saenz, Phaik Yeong Cheah, Rieke van der Graaf, Leslie Meltzer Henry, Anna C. Mastroianni

**Affiliations:** 1 Knowledge Management, Bioethics and Research, Pan American Health Organization, Washington, D.C, USA; 2 Mahidol Oxford Tropical Medicine Research Unit, Mahidol University, Bangkok, Thailand; 3 University Medical Center Utrecht, Utrecht University, Utrecht, Netherlands; 4 University of Maryland Carey School of Law, Baltimore, MD USA; 5 Johns Hopkins Berman Institute of Bioethics, Baltimore, MD USA; 6 University of Washington School of Law, Seattle, WA USA; 7 Nuffield Department of Medicine, University of Oxford, Oxford, UK

**Keywords:** Research ethics, Pregnancy research, Research regulation, Pregnancy, Medication

## Abstract

Scarce research with pregnant women has led to a dearth of evidence to guide medical decisions about safe and effective treatment and preventive interventions for pregnant women and their potential offspring. In this paper, we highlight three aspects of the landscape in which pregnant women are included or, more frequently, excluded from research: international ethics guidance, regional and national regulatory frameworks, and prevailing practices. Our paper suggests that, in some cases, regulatory frameworks can be more restrictive than international ethics guidance, and that even when regulations permit research with pregnant women, practical challenges—as well as the prevailing practices of stakeholders, such as ethics review committees and investigators—may lead to the generalized exclusion of pregnant women from research.

## Background

The underrepresentation—and often complete exclusion—of pregnant women from participation in clinical research has led to a dearth of evidence to guide medical decisions about safe and effective treatment and preventive interventions for pregnant women and their potential offspring [1, 2]. Despite that evidence gap, a recent review advocating for a better understanding of medication use during pregnancy concluded that, globally, pregnant women commonly take medication—whether prescription or over-the-counter [3]. Because metabolism changes during pregnancy, and some compounds cross the placental barrier, the absence of pregnancy-specific data for most medications means that pregnant women may be taking medications at doses that are ineffective and unsafe for both their own health and the health of their potential offspring [1]. There is a moral imperative to address this injustice [4].

In this paper, we highlight aspects of the landscape in which pregnant women are included or, more frequently, excluded from research. We discuss three key examples drawn from international ethics guidance, regional and national regulatory frameworks, and prevailing practices. For international ethics guidance, we focus on the 2016 edition of *International Ethical Guidelines for Health-related Research Involving Humans* by the Council for International Organizations of Medical Sciences (CIOMS). For regulatory frameworks, we focus on the laws and regulations of countries in the Americas. For prevailing practices, we provide an analysis based on our work experience in the Americas as investigators, members of ethics review committees, and advisors for national authorities in the development and revision of laws and regulations. We supplement this discussion of prevailing practices with an illustration of a practical challenge from Thailand. These examples suggest that, even when regulations and international ethics guidance permit research with pregnant women, practical challenges and the prevailing practices of stakeholders, such as ethics review committees and investigators, are factors that can perpetuate the exclusion of pregnant women from research.

## International ethics guidance

The most recent consensus statement of research ethics in global health is the 2016 revision to the *International Ethical Guidelines for Health-related Research Involving Humans*, first developed by CIOMS in 1982 and revised in 1993, 2002, and now 2016. CIOMS, a non-governmental organization, was established in 1949 by the World Health Organization (WHO) and the United Nations Educational, Scientific and Cultural Organization (UNESCO). Although not legally binding, the CIOMS Guidelines exert tremendous influence on country-specific legal approaches to the protection of research participants, particularly in low-resource settings. The revised CIOMS Guidelines provide detailed, comprehensive international ethics guidance for human subjects research, including updated guidelines pertaining to pregnant women. Notably, those revisions capture the consensus that “pregnant women must not be considered vulnerable simply because they are pregnant,” and that there is a duty to promote research designed to obtain knowledge relevant to the health needs of pregnant women ([5], p.58).

The revised CIOMS Guidelines permit research with pregnant women, provided certain conditions are met. Guideline 19, which specifically addresses research with pregnant women, makes distinctions based on the study’s potential benefit to the pregnant woman or the fetus. When research offers a potential benefit to either the pregnant woman or her fetus, risks must be minimized and outweighed by the prospect of potential individual benefit. When research does not offer potential individual benefits, “risks must be minimized and no more than minimal; and the purpose of the research must be to obtain knowledge relevant to the particular health needs of pregnant… women or their fetuses” ([5], p.71). A minor increase above minimal risk may be allowed when the social value of the study is compelling and the research cannot be conducted with non-pregnant women. Guideline 19 further stipulates that research involving pregnant women with the potential for harm to the fetus must be conducted only in settings where access to a safe, timely, and legal abortion can be guaranteed in the event that participation in research makes the pregnancy unwanted. However, Guideline 19 acknowledges that research ethics committees may permit research with compelling social value when this condition cannot be met [5]. Moreover, Guideline 19 specifies that, when enrolling in a study, the pregnant woman is the decision maker for any interventions that affect her, although this does not exclude the possibility of the woman consulting with the father of the fetus if she wishes to do so [5].

As explained in Guideline 18, which addresses research with women more broadly, women of childbearing potential must be given the opportunity to participate in research when eligible. Guideline 18 further adds that women who become pregnant during research should not be automatically removed from the study. If “there is no evidence on the basis of which a potential harm to fetus can be assumed, women … who become pregnant must be offered the option to continue or end their participation” ([5], p.70). These women could remain in the study for safety monitoring, even if administration of the study drug is stopped. If “a drug or biological product is known to be mutagenic or teratogenic, pregnant women must be removed from the study, and followed up and provided care through the duration of their pregnancy and delivery” ([5], p.70). This care includes access to diagnostic tests to reveal anomalies and referral for abortion if she wishes.

Given the significant revisions to the CIOMS Guidelines, successful implementation will require educating and training investigators and ethics review committees. Moreover, the revised CIOMS Guidelines call for reflection on the existing regulatory frameworks and their consistency with evolving ethics standards.

## Regulatory frameworks

### Latin America

While the CIOMS Guidelines support inclusion of pregnant women in research, existing laws in Latin America tend to be more restrictive [6]. The laws often permit only a subset of studies that would be considered ethical under the CIOMS Guidelines. It is not uncommon for pregnant women [7] or even women of childbearing age [8] to be referred to as a vulnerable population in regulatory frameworks of Latin American countries. Pregnant women are commonly permitted to participate in research for conditions that solely affect pregnant women or their fetuses—meaning that pregnancy is a requirement for inclusion in the study [9]. Several countries require that the father of the fetus also give consent to a pregnant woman’s participation in research unless circumstances make it impossible to do so [10–12]. For women of childbearing potential who are enrolled in research, the use of at least one contraception method is often required to ensure that they do not become pregnant during the study [13]. Women who become pregnant during a study are, as a rule, automatically removed from the study [11, 13, 14].

With few exceptions [10, 12], relevant laws in Latin America do not distinguish between studies that do or do not offer potential benefit for the pregnant woman or the fetus. As a result, the standard provisions for studies with no potential individual benefit are often applied to all cases of research during pregnancy. Definitions of minimal risk [14] and distinctions between levels of risk for research with pregnant women [9] are often absent, thus leading to a precautionary position that treats all levels of risk as equally unacceptable, which ultimately results in a categorical exclusion of pregnant women from all research that involves any risk—no matter how small—to either the pregnant woman or the fetus. While ethics review committees are tasked with reviewing and approving studies prior to their initiation, the regulatory frameworks often do not leave much leeway to the committees’ assessment on the basis of the specific characteristics of individual protocols because a share of research involving pregnant women is often already forbidden or restricted by the regulations. In a nutshell, a legitimate concern with the protection of pregnant women and their fetuses in research has prompted regulatory action and implementation that often forbids in *all* cases what might be unethical in *some* cases.

### United States

The United States permits pregnant women’s participation in research under conditions specified in federal regulations, known colloquially as the “Common Rule” and “Subpart B” [15, 16]. The Common Rule requires that research be reviewed and approved by a local ethics committee, known as an institutional review board (IRB), which is subject to national oversight. The Common Rule generally applies to research institutions and researchers that receive US federal funding or otherwise agree to abide by those directives. Subpart B specifies additional ethics requirements for research involving pregnant women, fetuses, and neonates, and is more limited in application than the Common Rule because not all government agencies have adopted Subpart B [17]. Both regulations may apply to international institutions and individuals (e.g., when they receive US government funding or otherwise agree to comply with the regulations). Pharmaceutical products (drugs, devices, and biologics) are subject to additional regulations promulgated by the Food and Drug Administration (FDA) [18–20]. The FDA does not have regulations specific to research with pregnant women, but has promulgated a number of guidelines on the topic [21].

Subpart B begins with a presumption that pregnant women, where pregnancy is defined as the period of time from implantation until delivery, may be included in research, provided certain conditions are met. According to Subpart B, the permissibility of research with pregnant women hinges on a judgment of the potential benefits and risks of the research. Approval of proposed research carrying no “prospect of direct benefit” to the woman or fetus requires that the risk to the fetus be judged “not greater than minimal” [16]. Fetal risk that exceeds that standard is permissible only when the proposed research offers a prospect of direct benefit to the pregnant woman, the fetus, or both. Notably, if the proposed research does not fit within either of those two parameters, Subpart B offers an additional mechanism at the national level for approval by the Secretary of Health and Human Services. To our knowledge, that process has never been used.

Lastly, consent of the woman is required in all cases. The consent of the father, however, is not mandatory unless the research has a prospect of direct benefit solely to the fetus (i.e., no such benefit to the pregnant woman herself). Exceptions to the paternal consent requirement are permissible when the father is unavailable, incompetent, temporarily incapacitated, or when the pregnancy resulted from incest or rape.

Although pregnant women are currently classified as “vulnerable” in the Common Rule, along with children, prisoners, and people with disabilities [15], a forthcoming revision removes that classification [22].

## Prevailing practices

Although U.S. regulations permit the inclusion of pregnant women in research under specified conditions, our experience has revealed that many stakeholders—including pharmaceutical companies, government and non-profit sponsors, researchers, IRBs, and individual regulators—continue to consider pregnancy as a near-automatic cause for exclusion, regardless of the harms imposed by exclusion or the likelihood of risks resulting from participation. Our work experience in Latin America has also revealed that, while the participation of pregnant women in research is already limited by national regulations, stakeholders similarly restrict their participation even further [23]. A variety of factors may contribute to such a cautious approach, including a protectionist mindset, concerns about liability, uncertainty about how to interpret relevant regulations, and lack of experience in assessing research with pregnant women [24]. Arguably, the prevailing distrust in biomedical research in Latin America also contributes to the exclusion of pregnant women from research.

The exclusion of pregnant women from clinical research is frequently justified as necessary to protect fetuses from unknown harms of in utero exposure to potentially teratogenic drugs [25, 26]. Stakeholders often articulate their protectionist stance as a desire to avoid “the next thalidomide,” a reference to the tragedy in which more than 10,000 children were born with birth defects resulting from in utero exposure to the drug [27, 28]. Notably, however, the thalidomide tragedy was not the result of pregnant women’s participation in research, but rather their use of a prescription drug that was never tested for safety or efficacy in pregnant women. Had pregnant women been included in drug trials, the magnitude of the tragedy arguably could have been mitigated, if not altogether averted [29].

Excluding pregnant women from clinical research is also a legal risk-mitigation strategy aimed at reducing stakeholders’ exposure to legal liability for fetal harms. Evidence from the 1970s that linked diethylstilbestrol (DES)—a drug prescribed to pregnant women to prevent miscarriage—with subsequent cancer in young women prenatally exposed to DES, amplified liability concerns by demonstrating that the period of legal risk was longer than previously understood [27].

Stakeholder uncertainty about the interpretation of relevant research regulations further contributes to the routine exclusion of pregnant women from clinical trials. Without additional guidance, for example, about how to understand what constitutes “minimal” risk to a fetus, stakeholders often opt for a conservative approach that excludes pregnant women altogether [24]. Taken together, those factors have created a situation in which pregnant women are so infrequently considered for inclusion in clinical research that few stakeholders have experience in balancing the potential benefits and risks of their participation.

As a consequence of pregnant women’s general exclusion from clinical research, medication risks are shifted to the clinical setting, where pregnant women receive neither the protection of formal risk monitoring nor the benefit of contributing to generalizable knowledge. More than 90% of medications approved by the FDA between 1980 and 2011 are reported to lack sufficient data to determine maternal and fetal risk [30]. The absence of safety and efficacy information does not, however, preclude physicians from prescribing—and pregnant women from taking—those medications “off-label” [27, 31]. Studies have revealed that as many as 93.9% of pregnant women in the United States take at least one medication [32, 33]. The slow accumulation of data resulting from post-marketing drug surveillance means that it can take as long as 27 years to gather sufficient data to identify medication-related risks to pregnant women and their potential offspring [30].

## Practical challenges

This landscape is additionally affected by various practical challenges that further hamper the inclusion of pregnant women in research globally. First, many countries still lack normative or regulatory frameworks for research with human subjects, which makes it even harder for stakeholders to decide on the inclusion of pregnant women in research. Additional difficulties are posed by problematic social and political conditions, which often impact access to healthcare, and by the ways in which women—and pregnant women specifically—are perceived in society. Further challenges are illustrated by the following case of research involving pregnant women in Thailand, many of whom are adolescents. Refugees, migrant workers, displaced people, and day migrants who cross into Thailand for employment converge in Thailand’s Tak province by the Myanmar border. Medical care is sought in Thailand due to limited access to medical care on the Myanmar side of the border. The Tak province is an area of multidrug-resistant malaria [34] where many important studies that have significantly influenced the recent changes in malaria therapy worldwide—i.e., use of artemisinin-based combination therapy (ACT)—took place. This includes a small number of studies conducted with pregnant women with malaria [35, 36]. Although these studies have contributed significantly to the evidence base for malaria in pregnancy, they have systematically excluded a significant proportion of pregnant women: those under the age of 18. Many adolescents are socially viewed as adults: they have their own households that are separate from their parents. Yet, because they have not reached the official age of majority or been legally emancipated, they cannot self-consent to participate in research. Because it is impractical or difficult for many of these pregnant adolescents to ask their parents to provide consent for their participation in research, they are frequently excluded from research even when they want to participate. Further, the exclusion of pregnant adolescents from research makes it difficult for some studies to achieve the necessary sample size.

## Conclusions

The inclusion of pregnant women in research is essential to offering evidence-based medical care for both pregnant women and their potential offspring. Ethics guidance that strongly promotes research during pregnancy does not suffice to ensure the inclusion of pregnant women in research because in some cases regulatory frameworks can be significantly more restrictive than such guidance. Even when regulations permit the research, prevailing practices and practical challenges can stymie research with pregnant women. It is therefore crucial to (a) raise awareness about the current dearth of evidence to guide medical decisions for pregnant women, and the moral imperative to redress this injustice; (b) provide research ethics training in accordance with international guidelines such as CIOMS; and (c) foster a dialogue with research communities, members of ethics review committees, research funders, regulatory agencies, and national health authorities to devise a plan to actively promote research with pregnant women.

## Abbreviations

ACT: Atemisinin-based combination therapy
CIOMS: Council for International Organizations of Medical Sciences
DES: Diethylstilbestrol
FDA: Food and Drug Administration
IRB: Institutional review board
UNESCO: United Nations Educational, Scientific and Cultural Organization
WHO: World Health Organization
